# Protective Effects of Crotonis Semen Extract against Sepsis through NF-κB Pathway Inhibition

**DOI:** 10.3390/ijms251810089

**Published:** 2024-09-19

**Authors:** Yo Sep Hwang, Hyang Ran Yoon, Hyo-Min Park, Jun-Pil Jang, Jun Hong Park, Seong-Hoon Park, Jong Seok Lim, Hee Jun Cho, Hee Gu Lee

**Affiliations:** 1 Immunotherapy Research Center, Korea Research Institute of Bioscience and Biotechnology, Yuseong-gu, Daejeon 34141, Republic of Korea; hys8520@kribb.re.kr (Y.S.H.); yhr1205@kribb.re.kr (H.R.Y.); wkd910222@kribb.re.kr (H.-M.P.); 2 Chemical Biology Research Center, Korea Research Institute of Bioscience and Biotechnology, Cheongju 28116, Republic of Korea; jpjang@kribb.re.kr; 3 Herbal Medicine Resources Research Center, Korea Institute of Oriental Medicine, Naju-si 58245, Republic of Korea; jhpark@kiom.re.kr; 4 Genetic and Epigenetic Toxicology Research Group, Korea Institute of Toxicology, Daejeon 34114, Republic of Korea; seonghoon.park@kitox.re.kr; 5 Department of Biological Science and the Cellular Heterogeneity Research Center, Research Institute of Women’s Health, Sookmyung Women’s University, Seoul 04310, Republic of Korea; jslim@sookmyung.ac.kr; 6 Department of Biomolecular Science, KRIBB School of Bioscience, Korea University of Science and Technology (UST), Yuseong-gu, Daejeon 34113, Republic of Korea

**Keywords:** crotonis semen, anti-inflammation, sepsis, macrophages, NF-κB

## Abstract

Sepsis is an inflammatory condition causing organ failure due to an uncontrolled immune response to infection and remains a significant challenge. Crotonis Semen has displayed various pharmacological effects, yet its potential in protecting against sepsis and the mechanisms involved remains largely unclear. Here, we explored the antiseptic properties of Crotons Semen extract (CSE) in both LPS-stimulated J774 macrophages and mice subjected to sepsis through Cecal ligation and Puncture (CLP) or LPS induction. We found that CSE enhanced survival rates in mouse models with acute sepsis induced by CLP operation and LPS injection. Administering CSE also reduced levels of enzymes indicating organ damage, such as aspartate aminotransferase (AST), alanine aminotransferase (ALT), and creatine kinase (CK), in septic mice. Furthermore, CSE lowered the serum levels of inflammatory mediators and cytokines, such as NO, TNF-α, IL-1β, and IL-6, in septic mice. In LPS-stimulated J774 macrophages, CSE reduced the expression of pro-inflammatory proteins, including iNOS and COX-2. Moreover, CSE inhibited the phosphorylation of IκBα and IKK, key components of the NF-κB signaling pathway, thereby reducing inflammatory mediators and cytokines. These results demonstrate CSE’s protective effects against sepsis through NF-κB pathway disruption, indicating its potential as a therapeutic option for acute inflammatory conditions.

## 1. Introduction

Sepsis is a severe and widespread inflammatory response that arises when the immune system reacts intensely to an infection caused by bacteria, viruses, or other pathogens. In 2017, the World Health Organization identified sepsis as a major global health concern because of its widespread occurrence and high mortality rates. It is defined by the presence of microbial byproducts or toxins in the bloodstream, leading to a systemic inflammatory response syndrome [1,2,3]. This reaction leads to the release of pro-inflammatory cytokines, which initiate a cascade effect, producing more cytokines and resulting in cellular and organ damage. The cascade of cytokines is responsible for the various local and systemic symptoms associated with the infection. If this process exceeds a certain threshold, it can progress to a severe condition known as “sepsis”, which has a profound impact on both morbidity and mortality [4,5]. Therefore, managing excessive immune responses with anti-inflammatory treatments may be crucial in reducing the mortality associated with sepsis.

Macrophages are essential to the pathophysiology of sepsis, as they secrete various pro-inflammatory factors such as TNF-α, IL-1, IL-6, and reactive nitrogen and oxygen species, which are crucial for fighting pathogens and tumors [6,7]. When an infection occurs, inflammatory monocytes circulate and differentiate into macrophages, working alongside resident macrophages in the affected tissues. Upon recognizing pathogen-associated molecular patterns through pathogen recognition receptors like Toll-like receptors (TLRs), macrophages become activated and release inflammatory mediators, such as TNF-α, IL-1, IL-6, and nitric oxide (NO). These substances help combat infections and stimulate adaptive immune responses [8,9]. However, if this inflammatory response becomes excessive, it can lead to a cytokine storm, potentially advancing to severe sepsis [10].

Crotonis Semen (CS), known scientifically as the seeds of the *Croton tiglium* plant, is also referred to as *Croton* seed or *Tiglium* seed. This plant holds a significant place in traditional medicine practices throughout Asia, Africa, and Latin America due to its various therapeutic uses. The seeds of *Croton tiglium* contain a complex array of bioactive compounds, and the extracts derived from these seeds, termed CS extracts (CSEs), have been shown to possess a range of pharmacological properties [11,12]. CSE exhibits a variety of beneficial effects, including larvicidal activity, which makes it effective against larvae of certain pests [13]. Additionally, CSE has been shown to have antinociceptive properties, meaning it can help reduce pain perception [14]. The extract also offers neuroprotective effects, suggesting it can protect nerve cells from damage [15], and possesses analgesic properties, providing relief from pain [16]. Furthermore, CSE has demonstrated antitumor activity, indicating its potential in combating cancerous growths [17], and antifungal activity, which could be useful in treating fungal infections [18]. Despite these diverse applications, the precise impact of CSE on sepsis has not been thoroughly investigated.

This study aimed to investigate the potential of CSE in treating sepsis using mice models with sepsis induced by CLP and LPS. Additionally, we examined the mechanisms behind CSE’s anti-septic effects in LPS-stimulated macrophages, evaluating its potential as a treatment for sepsis.

## 2. Results

### 2.1. CSE Improves Survival and Reduces Inflammation in Mouse Models of Sepsis Induced by LPS

**Figure 1:**
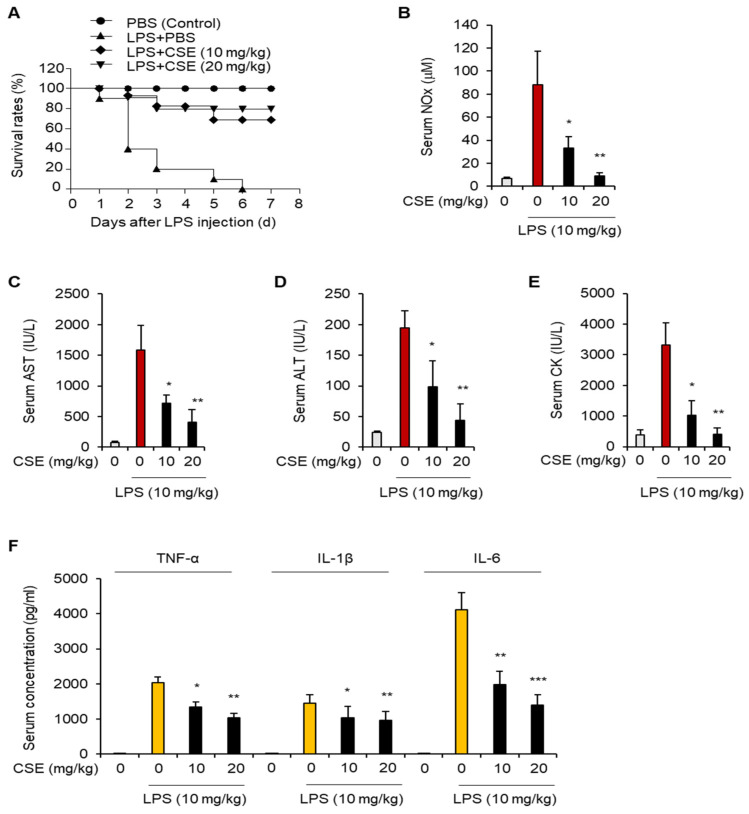
The effect of CSE on survival in mice with LPS-induced septic shock. Mice were given oral doses of CSE (10 or 20 mg/kg) or PBS for 7 days prior to LPS injection (*n* = 10 per group). Mice receiving PBS alone served as negative controls. (**A**) Survival rate following LPS administration. (**B**–**F**) After the 7-day treatment, mice were injected with LPS, and serum was collected 12 h later. The levels of NO (**B**), AST (**C**), ALT (**D**), CK (**E**), and pro-inflammatory cytokines including TNF-α, IL-1β, and IL-6 (**F**) were assessed in the serum of LPS-induced septic mice. * *p* < 0.05, ** *p* < 0.01, *** *p* < 0.001.

We first assessed the protective effects of CSE in a mouse model of septic shock induced by LPS injection. Mice were administered CSE orally for 7 days before receiving the LPS injection, and their mortality was assessed. The survival rates of mice treated with CSE showed a significant dose-dependent improvement, compared to those receiving LPS alone (Figure 1A). Additionally, in the PBS control group, LPS treatment led to a marked increase in serum levels of NO, AST, ALT, and CK, but these elevations were reduced by CSE administration (Figure 1B–E). CSE treatment also lowered the elevated serum levels of TNF-α, IL-1β, and IL-6 induced by LPS (Figure 1F). These findings demonstrate that CSE improves survival and reduces liver damage and inflammation in mice with LPS-induced septic shock.

### 2.2. CSE Enhances Survival Rates in CLP-Induced Septic Mouse Models

**Figure 2:**
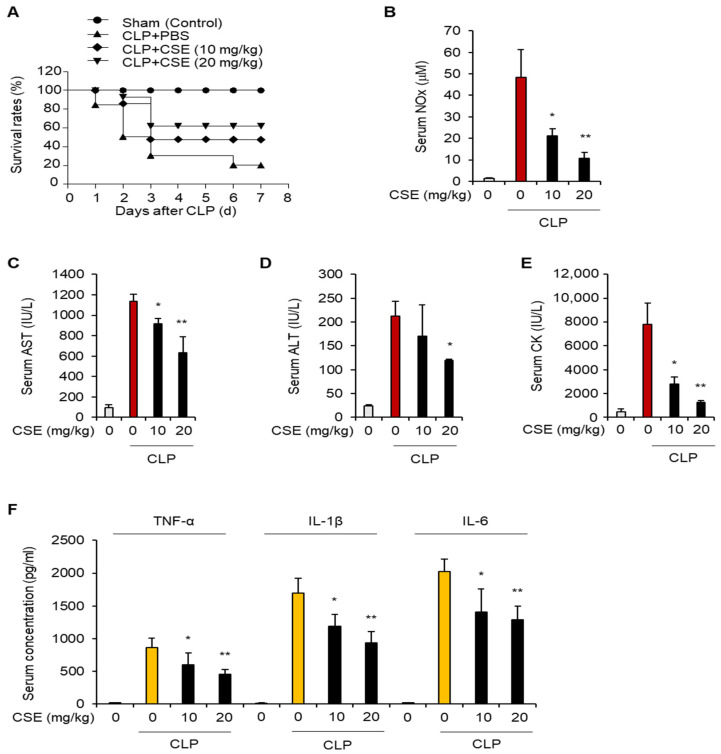
The effect of CSE on survival in mice with CLP-induced septic shock. Mice were given oral doses of CSE (10 or 20 mg/kg) or PBS for 7 days before undergoing CLP surgery (n = 10 per group). Sham-operated mice served as negative controls. (**A**) Survival rates after CLP surgery were monitored. (**B**–**F**) Mice received daily oral doses of CSE (10 or 20 mg/kg) or PBS for 7 days before CLP surgery (n = 10 per group), with sham-operated mice as negative controls. Serum samples were collected 12 h post-CLP surgery to assess levels of NO (**B**), AST (**C**), ALT (**D**), and CK (**E**), and indicated pro-inflammatory cytokines (**F**) in CLP-induced septic mice. * *p* < 0.05, ** *p* < 0.01.

We established a sepsis model using CLP surgery in addition to the LPS-induced sepsis model to better simulate real-world infections and subsequent sepsis from microbial transfer [19]. We then assessed the impact of CSE on this CLP model. Mice were given CSE (10 or 20 mg/kg) or PBS daily for seven days before undergoing CLP. Survival rates were monitored for seven days post-CLP surgery. CSE administration significantly improved survival rates in the CLP mice relative to the CLP-only group (Figure 2A). Following CLP surgery, serum levels of NO, AST, ALT, and CK significantly rose in the PBS-fed group, but these increases were reduced with CSE treatment (Figure 2B–E). Additionally, CSE administration lowered elevated serum levels of TNF-α, IL-1β, and IL-6 caused by CLP surgery (Figure 2F). These results further support that CSE enhances survival and reduces liver damage and inflammation in mice with CLP-induced septic shock.

### 2.3. CSE Inhibits Pro-Inflammatory Responses Induced by LPS in J774 Macrophages

**Figure 3:**
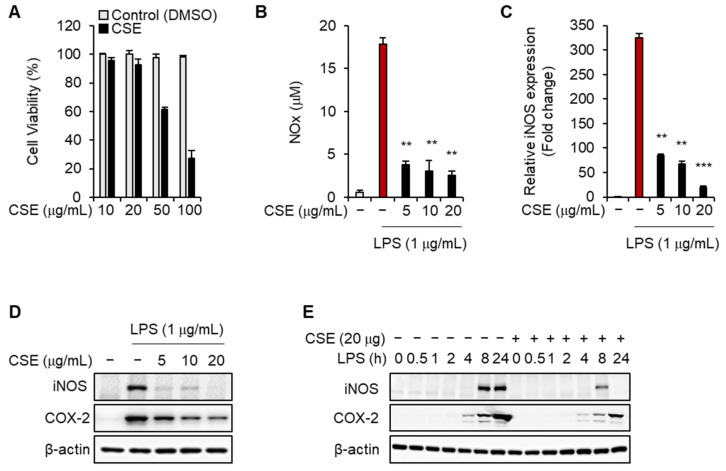
The effects of CSE on the expression of inflammatory mediators induced by LPS in macrophage cells. (**A**) J774 cells were treated with various concentrations of CSE for 24 h, and cell viability was assessed. (**B**–**D**) J774 cells were pre-treated with different concentrations of CSE for 1 h prior to LPS exposure and then cultured for 24 h. (**B**) NO levels in culture media were measured. (**C**) mRNA levels of iNOS were evaluated by qRT-PCR. (**D**) Protein levels of iNOS and COX-2 were analyzed using WB. (**E**) J774 cells were treated with 20 µg/mL CSE before LPS exposure at different time points. Protein levels of iNOS and COX-2 were analyzed using WB. ** *p* < 0.01, *** *p* < 0.001.

Given that macrophages play a central role in various pathophysiological aspects of sepsis, exploring mechanisms that target these cells is of significant importance [6,7]. Thus, we investigated the anti-inflammatory effects of CSE specifically in macrophages. We first determined the optimal concentration of CSE to assess its impact on LPS-induced inflammation in J774 macrophages. While concentrations of 10 or 20 µg/mL CSE did not affect cell survival, higher doses of 50 or 100 µg/mL led to a 40% and 70% reduction in J774 macrophage survival, respectively (Figure 3A). Pre-treatment with 5, 10, or 20 µg/mL CSE followed by stimulation with 1 µg/mL LPS significantly decreased NO production (Figure 3B). Additionally, LPS treatment increased the expression of iNOS mRNA and protein, which was reduced by CSE treatment in a dose-dependent manner (Figure 3C,D). CSE also decreased elevated COX-2 levels associated with inflammation, with a dose-dependent effect observed (Figure 3D). Furthermore, CSE treatment mitigated the increases in iNOS and COX-2 levels that occurred at different time points following LPS exposure (Figure 3E).

**Figure 4:**
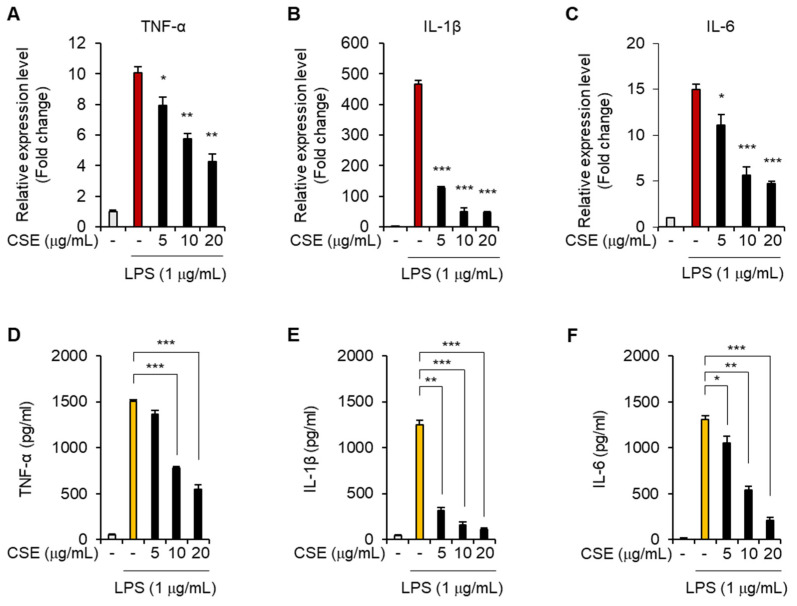
Effect of CSE on pro-inflammatory cytokine production in macrophages stimulated by LPS. (**A**–**F**) J774 macrophages were pre-treated with indicated concentrations of CSE for 1 h before LPS exposure and then cultured for 24 h. (**A**–**C**) mRNA levels of TNF-α, IL-1β, and IL-6 were evaluated by qRT-PCR. (**D**–**F**) Secretion levels of TNF-α, IL-1β, and IL-6 were analyzed using ELISA. * *p* < 0.05, ** *p* < 0.01, *** *p* < 0.001.

When macrophages release excessive amounts of pro-inflammatory cytokines such as TNF-α, IL-1, and IL-6 in an uncontrolled manner, it can lead to a severe inflammatory response known as a cytokine storm, which can progress to severe sepsis [10]. To investigate whether CSE could mitigate this inflammatory response, we exposed J774 macrophages to LPS after pre-treating them with CSE. We assessed the impact of CSE on the production of inflammatory cytokines, including TNF-α, IL-1β, and IL-6. LPS exposure significantly increased the levels of these cytokines in the culture media. However, treatment with CSE notably reduced the mRNA levels of TNF-α, IL-1β, and IL-6 (Figure 4A–C). Further confirmation through ELISA demonstrated that CSE reduced the production of TNF-α, IL-1β, and IL-6 in a dose-dependent manner following LPS stimulation (Figure 4D–F). These findings indicate that CSE effectively inhibits the production of pro-inflammatory cytokines induced by LPS in macrophages.

### 2.4. CSE Attenuates the Activation of NF-κB by LPS in J774 Cells

**Figure 5:**
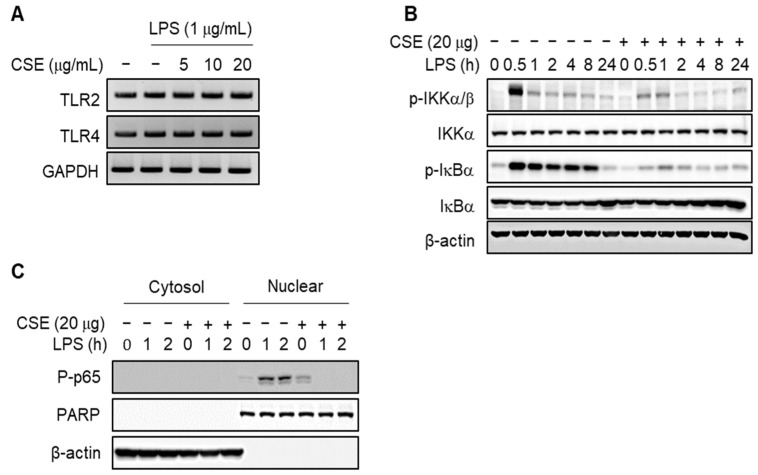
Inhibitory effect of CSE on NF-κB activation. J774 macrophages were exposed to indicated specific concentrations of CSE 1 h prior to LPS exposure, followed by a 24-h culture period. (**A**) Effects of CSE on TLR2 and TLR4 mRNA expression. (**B**) J774 macrophages were exposed to 20 µg/mL CSE for 1 h and then treated with LPS at the indicated time points. The effect of CSE on the phosphorylation of IKKα/β and IκBα was verified based on the duration of LPS treatment. (**C**) CSE’s influence on the nuclear translocation of NF-κB p65 was observed, with PARP and β-actin serving as loading controls for the nuclear and cytosolic fractions, respectively.

Since NF-κB regulates the expression of various inflammatory mediators and cytokines [20,21], we explored its role in the anti-inflammatory effects of CSE. Initially, CSE did not alter the mRNA levels of TLR2 and TLR4 (Figure 5A). However, CSE treatment significantly decreased the phosphorylation of IKKα/β and IκB induced by LPS in J774 macrophages (Figure 5B). Furthermore, CSE notably inhibited the nuclear translocation of p65 triggered by LPS (Figure 5C), suggesting that CSE mitigates the inflammatory response to LPS by inhibiting NF-κB activation in macrophages.

### 2.5. LC-MS Base Peak Chromatogram of Ethanol Extracts of Crotonis Semen

**Figure 6:**
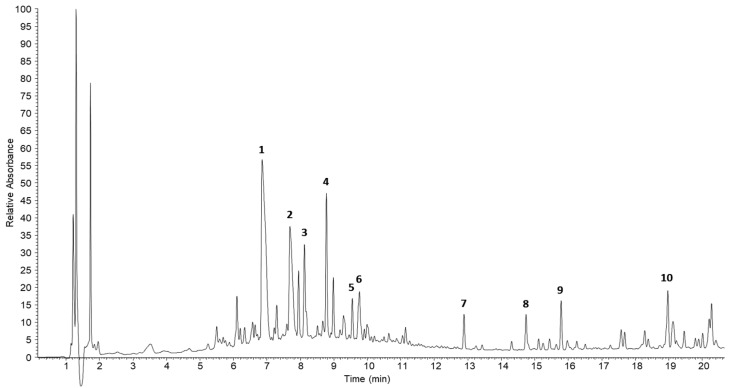
LC-MS chromatogram of the 70% ethanol extracts of *Croton tiglium*. Thaliporphine (1), glaucine (2), crotoxide A (3), megalocarpodolide D (4), laurelliptin (5), crotignoid K (6), crotophorbolon-17-acetat (7), 12-*O*-tiglylphorbol-13-acetate (8), 12-*O*-tigloylphorbol-13-(4*Z*,7*Z*)-decadienoate (9), 12-*O*-dodecanoylphorbol-13-acetate (10).

The analysis of 70% ethanol extracts of Crotonis Semen was conducted using LC-MS selected ion chromatography on a reversed-phase column with a linear gradient from 5% to 100% aqueous CH_3_CN containing formic acid over 15 min. By comparing the retention times with those of reference compounds obtained from the plant, 10 major peaks were identified (Figure 6).

## 3. Discussion

Sepsis is a critical and potentially fatal condition caused by an extreme immune response to infection. The body’s attempt to combat an infection can sometimes escalate into widespread inflammation, leading to serious complications and damage to multiple organs [22]. Treating sepsis poses significant challenges, such as difficulties in early diagnosis, antibiotic resistance, extensive organ damage, and the need for complex treatment regimens, along with various complications and long-term effects. Despite ongoing research and medical advances, effective treatments for sepsis remain elusive [23,24]. Natural products offer a promising solution due to their diverse biological activities, including antibacterial, anti-inflammatory, and immunomodulatory properties [25,26]. Crotonis Semen extract (CSE) is known for its broad pharmacological effects, including larvicidal [13], antinociceptive [14], neuroprotective [15], analgesic [16], antitumor [17], and antifungal [18] activities. However, its potential in sepsis treatment has not been previously investigated. This study demonstrates that CSE improves survival in sepsis models by reducing the excessive inflammatory responses in mice subjected to sepsis caused by CLP surgery and LPS injection.

Based on the LC-MS chromatogram analysis of 70% ethanol extracts from Croton tiglium, several key components have been identified, including Thaliporphine, Glaucine, Crotoxide A, Megalocarpodolide D, Laurelliptin, Crotignoid K, Crotophorbolon-17-acetate, 12-*O*-Tiglylphorbol-13-acetate, 12-*O*-Tigloylphorbol-13-(4*Z*,7*Z*)-decadienoate, and 12-*O*-Dodecanoylphorbol-13-acetate (Figure 6). Existing research indicates that Thaliporphine can mitigate endotoxin-induced circulatory failure and reduce mortality in rat models [27]. Glaucine has been reported to significantly decrease cell infiltration, cartilage degradation, and bone erosion in arthritis models [28]. The presence of various phorbol-type compounds in Croton tiglium extracts, such as crotophorbolon-17-acetate, 12-*O*-tiglylphorbol-13-acetate, 12-*O*-tigloylphorbol-13-(4Z,7Z)-decadienoate, and 12-*O*-dodecanoylphorbol-13-acetate, is noteworthy (Figure 6). Research has shown that phorbol 12-myristate 13-acetate (PMA), a phorbol derivative, can enhance autophagy through a NET-dependent mechanism in sepsis mouse models, leading to improved survival rates [29]. Consequently, the anti-inflammatory properties of Croton tiglium extracts during sepsis are likely attributed to these diverse pharmacological activities. Identifying the specific active compounds within these extracts could facilitate the isolation and synthesis of individual components, providing a more comprehensive understanding of their therapeutic potential.

Sepsis results in widespread tissue damage, as evidenced by significant increases in biochemical markers [30,31]. AST and ALT are transaminases that serve as indicators of liver damage and are commonly used to evaluate liver function in clinical practice [32]. CK is a key enzyme involved in energy regulation, and it is crucial for intracellular energy transfer, muscle contraction, and ATP regeneration [33]. In this study, we assessed the effect of CSE on organ damage induced by sepsis by measuring biochemical markers, such as AST, ALT, and CK. Our results revealed a significant reduction in serum levels of AST, ALT, and CK in mice with CLP- and LPS-induced sepsis following CSE treatment. Therefore, CSE could play a protective role against liver injury and skeletal muscle damage.

During sepsis, iNOS increases NO production as part of the immune response to pathogens. NO plays a dual role in sepsis: initially, it aids in immune regulation by acting as a vasodilator, modulating inflammation, and helping to eliminate pathogens. However, excessive NO can have detrimental effects, such as contributing to vasodilation, vascular dysfunction, low blood pressure, and tissue damage, which exacerbate septic shock. Thus, monitoring and managing NO levels in septic patients is crucial due to its dual role in both protective and harmful pathways [34,35,36]. Additionally, sepsis is characterized by increased release of pro-inflammatory cytokines, such as IL-1β, IL-6, and TNF-α. These cytokines facilitate cell signaling and drive inflammatory responses, playing a key role in both the early and ongoing inflammatory phases of sepsis. The overproduction of these cytokines is strongly associated with patient outcomes, suggesting that targeting and suppressing these inflammatory markers could be a promising therapeutic strategy for sepsis [37,38]. In our study, we observed a significant increase in NO levels and pro-inflammatory cytokines IL-1β, IL-6, and TNF-α in the serum of mice with CLP- and LPS-induced sepsis. Notably, treatment with CSE substantially reduced these elevated levels. These results highlight CSE’s potential to modulate excessive NO and pro-inflammatory cytokine production in sepsis. Consequently, our study suggests that CSE could be an effective therapeutic approach for managing sepsis.

Macrophages release various pro-inflammatory substances, including TNF-α, IL-1, IL-6, and reactive nitrogen and oxygen species, in response to external pathogens. While these cytokines are crucial for combating infections and tumors, uncontrolled inflammatory responses can lead to a cytokine storm and severe sepsis [10]. Thus, targeting inflammatory cytokines presents a potential strategy for treating sepsis. In this study, CSE was shown to enhance the survival rate of mice with sepsis induced by CLP and LPS. It also alleviated liver damage and improved the condition of skeletal muscle tissue, as indicated by biochemical markers. Consequently, CSE reduced levels of inflammatory cytokines and factors associated with sepsis, as anticipated. Further investigation into the anti-inflammatory effects of CSE, both in vitro and in vivo, revealed its ability to effectively suppress the production of IL-1β, IL-6, and TNF-α in LPS-stimulated macrophages. Additionally, CSE reduced iNOS, COX-2, and NO levels induced by LPS in macrophages (Figure 3). These results suggest that CSE offers protective benefits against septic shock by moderating the excessive inflammation triggered by macrophages during septic conditions.

The NF-κB signaling pathway is well established as a critical component in the development of sepsis. It regulates the activation of numerous genes associated with inflammatory responses [39,40]. NF-κB activation begins with the phosphorylation of the IκB protein by the IκB kinase (IKK) complex, which is activated by a range of microbial pathogens. This phosphorylation leads to the degradation of IκBα, permitting NF-κB to translocate into the nucleus where it governs the expression of inflammatory genes, including NO, iNOS, COX-2, IL-1β, IL-6, and TNF-α. Targeting IKK and IκBα has shown potential in reducing excessive inflammation and in alleviating cardiac and liver dysfunctions associated with sepsis [41,42]. Our study found that CSE effectively inhibited the production of inflammatory mediators and cytokines triggered by LPS in macrophages. We further explored its effects on LPS-induced NF-κB activation and found that CSE obstructed the phosphorylation of IKK and IκBα, which in turn prevented the nuclear translocation of the p65 NF-κB subunit. These results indicate that CSE reduces the expression of iNOS, COX-2, and pro-inflammatory cytokines, such as IL-1β, IL-6, and TNF-α, which are regulated by NF-κB in LPS-stimulated macrophages. In conclusion, these findings demonstrate that CSE may play a crucial role in exerting anti-septic and anti-inflammatory effects, at least in part, by inhibiting the NF-κB signaling pathway.

## 4. Materials and Methods

### 4.1. Preparation of ACSEs

We purchased lyophilized powder of CSE prepared from 70% ethanol extract (KOC-70E-370) from KOC Biotech Co. (Daejeon, Republic of Korea). The CSE powder was dissolved in 10% DMSO (Sigma, St. Louis, MO, USA).

### 4.2. Cell Culture

The J774A.1 cell line was acquired from the ATCC (Rockville, MD, USA) and cultured in Dulbecco’s modified Eagle’s medium supplemented with 10% fetal bovine serum (HyClone, Logan, UT, USA) and antibiotics (Gibco-BRL, New York, NY, USA). The cells were maintained in a humidified incubator at 37 °C with 5% CO_2_. For experimental treatments, J774A.1 cells stimulated with LPS were incubated with various concentrations of CSE for different time periods.

### 4.3. Animals Studies

C57BL/6 mice (male, aged 7–8 weeks) were obtained from the Korea Research Institute of Bioscience and Biotechnology (KRIBB; Cheongju, Republic of Korea) and maintained at the animal experiment center of KRIBB (Daejeon, Republic of Korea) with a temperature-controlled room (22 ± 2 °C) and 12 h light/dark cycle (lights on at 8 a.m.). All animal experiments were approved by the Institutional Animal Care and Use Committee of KRIBB and were performed following KRIBB’s institutional guidelines (KRIBB-AEC-21079). The sepsis models for each mouse were established as described below: 

LPS-induced septic shock model: Mice were assigned to one of four groups (control, LPS, CSE-10, CSE-20; n = 10 per group) and were fasted overnight. The CSE groups (CSE-10 and CSE-20) received 10 and 20 mg/kg of CSE dissolved in phosphate-buffered saline (PBS; WELGENE, Gyeongsan, Republic of Korea, 100 µL/mouse), respectively, while the vehicle control group (LPS) received PBS (100 µL/mouse) via oral gavage for 7 days. On the following day, mice were injected intraperitoneally with LPS (10 mg/kg) to induce sepsis and observe the resulting lethality. The control group was given PBS instead of LPS to serve as a negative control. Mice survival was monitored for 7 days after LPS administration. 

CLP-induced septic shock model: Mice were assigned to one of four groups (sham, CLP, CSE-10, CSE-20; n = 10) and fasted overnight. The CSE groups (CSE-10 and CSE-20) received 10 and 20 mg/kg of CSE dissolved in phosphate-buffered saline (PBS; 100 µL/mouse), respectively, while the vehicle control group (CLP) received PBS (100 µL/mouse) via oral gavage for 7 days. On the following day, mice were anesthetized with an intraperitoneal injection of avertin (Sigma, St. Louis, MO, USA, 500 mg/kg). Then, CLP surgery was performed as previously described [43]. Mouse survival was monitored for 7 days following the CLP surgery.

### 4.4. Blood Sample Collection

Mice were administered either 10 or 20 mg/kg of CSE or control PBS orally for 7 days. The following day, the mice (n = 5) were either injected with LPS or underwent CLP surgery. A total of 12 h after the LPS injection or CLP surgery, blood samples were collected from the mice as previously described [44].

### 4.5. Cell Viability

Cell viability was analyzed using the water-soluble tetrazolium salt (WST)-1 assay (Roche, Basel, Switzerland) according to the manufacturer’s instructions. A total of 10,000 cells were plated in 96-well plates and allowed to adhere overnight. The following day, the cells were treated with various concentrations of CSE (10, 20, 50, and 100 µg/mL) for 24 h. After treatment, 10 µL of WST-1 reagent was added to each well, and the plates were incubated for 1 h. The absorbance at 450 nm was then measured using a microplate reader (Molecular Devices, Sunnyvale, CA, USA) to determine the conversion of WST-1 reagent to chromogenic formazan, reflecting cell viability.

### 4.6. NO Measurements

The NO levels were determined by analyzing 100 µL of culture supernatants or serum samples following a previously described method [44]. Briefly, the samples were combined with an equal volume of Griess reagent and incubated for 10 min. For serum samples, measurements were conducted after appropriate dilution with sample dilution buffer (R&D Systems, Minneapolis, MN, USA). Absorbance was measured at 562 nm using a microplate reader. The NO levels were determined by comparing the absorbance values to a standard curve generated with sodium nitrite.

### 4.7. Enzyme-Linked Immunosorbent Assay (ELISA)

The cytokine levels were measured using the mouse TNF-α (DY-410), IL-1β (DY-401), and IL-6 (DY-406) DuoSet ELISA kits. All ELISA kits were sourced from R&D Systems and were employed according to the standard protocols described previously [45].

### 4.8. Western Blotting

Cells were lysed in RIPA buffer (Sigma, St. Louis, MO, USA), and the protein concentrations were determined using the BCA assay (IntronBiotechnology, Seong-Nam, Republic of Korea). Protein lysates were then separated by 8–12% SDS-PAGE. After electrophoresis, the proteins were transferred to PVDF membranes. The membranes were blocked with 5% skim milk and probed with the following antibodies: iNOS (SC-650, SCBT, Dallas, TX, USA), Cox2 (Cell Signaling Technology, CST, Danvers, MA, USA, #12282), pIKKα/β (CST, #2697), IKKα (CST, #61294), pIκBα (CST, #2859), IκBα (CST, #9242), p-p65 CST(#3033), PARP (CST, #9532), and β-actin (SCBT, SC-47778). Next, appropriate HRP-conjugated secondary antibodies were incubated for 40 min at room temperature. The membranes were visualized using a chemiluminescent HRP substrate (Milipore, Billerica, MA, USA).

### 4.9. Quantitative RT-PCR (qRT-PCR) 

Total RNA was extracted from cells using an RNA extraction kit (Biofact, Daejeon, Republic of Korea). cDNA was synthesized from total RNA using oligo dT primer and a GoScriptTM Reverse Transcription System (Promega, Madison, WI, USA). qRT-PCR was performed using AccuPower 2X Greenstar^TM^ qPCR Master Mix (Bioneer, Daejeon, Republic of Korea) and StepOnePlus Real-Time PCR (Thermo Fisher Scientific, Rockford, IL, USA). Gene expression was calculated using the 2^−ΔΔCt^ method. All samples were tested in triplicate. GAPDH was used as endogenous control. The primer sequences are listed in Table S1.

### 4.10. LC-MS Analysis

For liquid chromatography–mass spectrometry (LC-MS) analysis, we employed an LTQXL linear ion trap mass spectrometer from Thermo Scientific (Rockford, IL, USA), equipped with an electrospray ionization (ESI) source. This was coupled with a rapid separation LC system (Ultimate 3000, Thermo Scientific) using a Waters HSS T3 column (Waters, Milford, MA, USA, 2.1 × 150 mm, 2.5 µm).

### 4.11. Statistical Analysis

All statistical analyses were performed using GraphPad Prism 9 (GraphPad Inc., San Diego, CA, USA). Data are presented in terms of the mean ± standard deviation. To assess differences between groups, an unpaired two-tailed Student’s *t*-test was used. Statistical significance was defined by a *p*-value less than 0.05, indicating meaningful differences between the groups.

## 5. Conclusions

**Figure 7:**
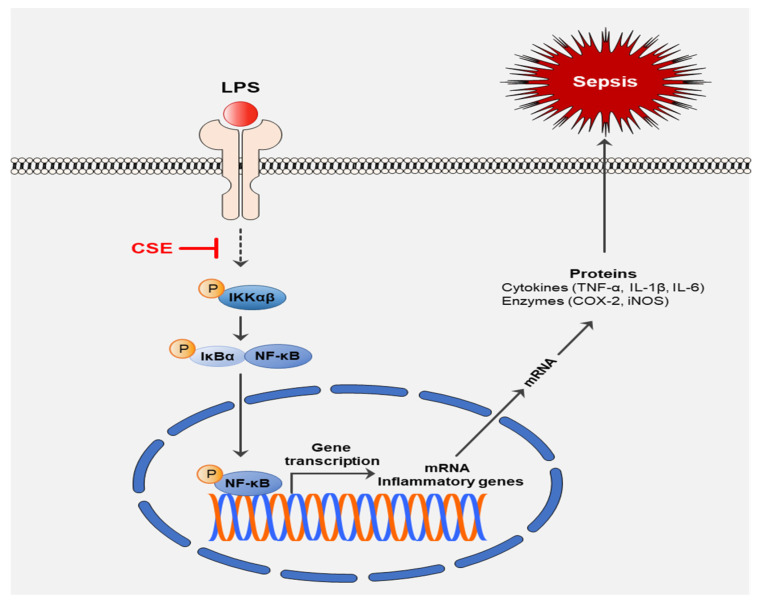
The protective effects of CSE on LPS-induced septic shock.

In summary, our study is the first to demonstrate the anti-inflammatory effects of CSE and elucidate its mechanisms. These results underscore CSE’s potential in alleviating septic shock by reducing inflammatory mediator production through the inhibition of the NF-κB pathway (Figure 7). Although further research is needed to explore CSE’s effects on various sepsis-related factors, our results indicate that CSE could be a promising therapeutic candidate for sepsis.

## Data Availability

The original contributions presented in this study are included in the article/Supplementary Materials; further inquiries can be directed to the corresponding authors.

## Supplementary Materials

The following supporting information can be downloaded at: https://www.mdpi.com/article/10.3390/ijms251810089/s1.
