# Supplementary material for: Protective Effects of Crotonis Semen Extract against Sepsis through NF-κB Pathway Inhibition
Source: Int J Mol Sci. 2024 Sep 19;25(18):10089. doi: 10.3390/ijms251810089 (PMC11432241; doi:10.3390/ijms251810089)
Supplement: Supplementary file 1 [file ijms-25-10089-s001.zip › ijms-3200660-supplementary.pdf]

Table S1. DNA oligomers list used in RT-PCR.

Gene Name	(5') Forward Primers (3')	(5') Reverse Primers (3')
**TLR2**	TTGCTCCTGCGAACTCCTAT	GCTTTCTTGGGCTTCCTCTT
**TLR4**	GCTTTCACCTCTGCCTTCAC	AGGCGATACAATTCCACCTG
**iNOS**	CAAGCACCTTGGAAGAGGAG	AAGGCCAAACACAGCATACC
**COX2**	GCTGTACAAGCAGTGGCAAA	TTCTGCAGCCATTTCCTTCT
**TNF-α**	ACGGCATGGATCTCAAAGAC	TGAGATAGCAAATCGGCTGAC
**IL-1β**	GAGTGTGGATCCCAAGCAAT	CTTGTGCTCTGCTTGTGAGG
**IL-6**	CTGATGCTGGTGACAACCAC	TCCACGATTTCCCAGAGAAC
**GAPDH**	ACCCAGAAGACTGTGGATGG	ACACATTG GGGGTAGGAACA
